# Supplementary figures and images for: The effects of zinc sulfate on mycelial enzyme activity and metabolites of Pholiota adiposa
Source: PLoS One. 2023 Dec 21;18(12):e0295573. doi: 10.1371/journal.pone.0295573 (PMC10735028; doi:10.1371/journal.pone.0295573)

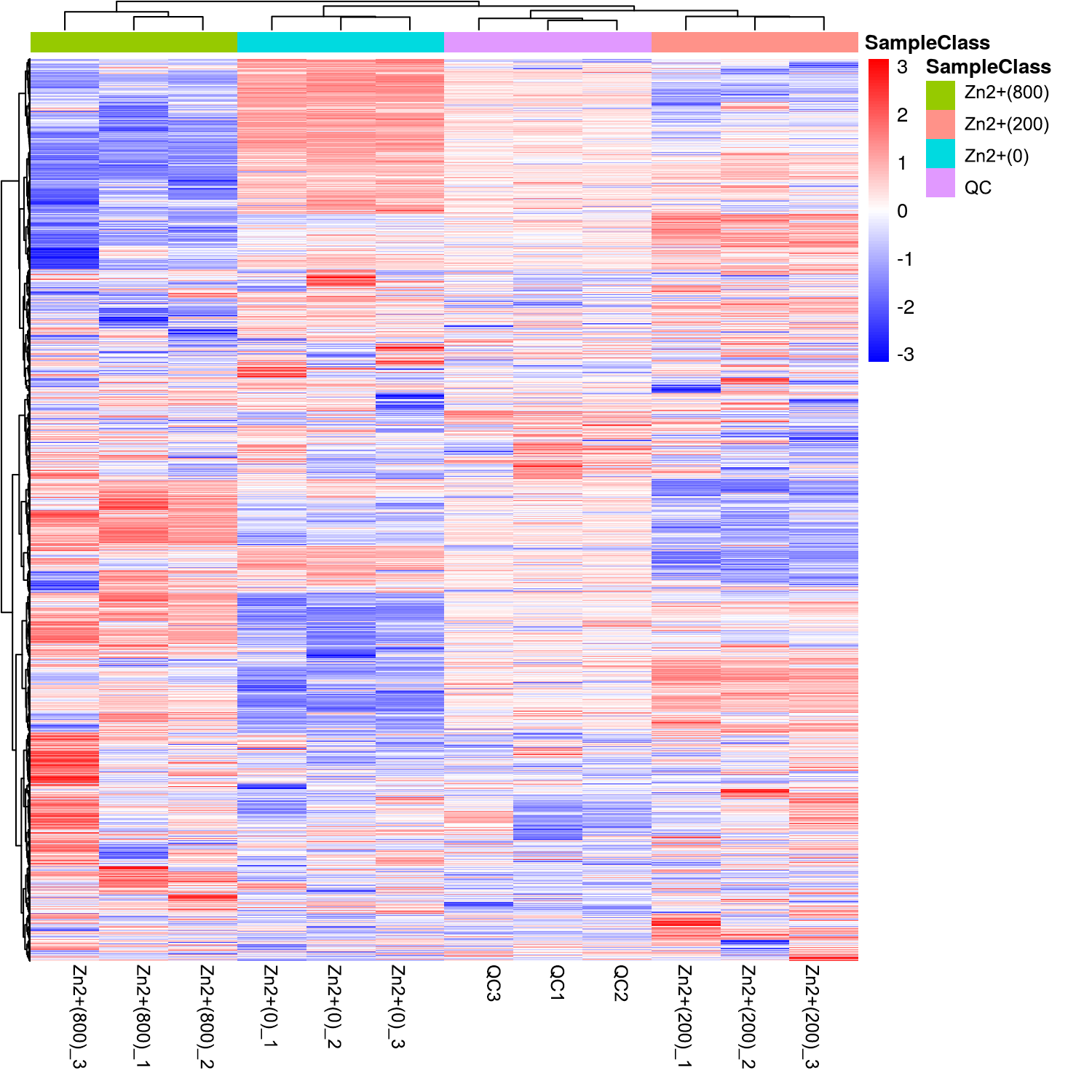

Supplement: S2 Data — (ZIP) [file pone.0295573.s002.zip › Metabonomic data/combine.intensity.heatmap.cluster.png]

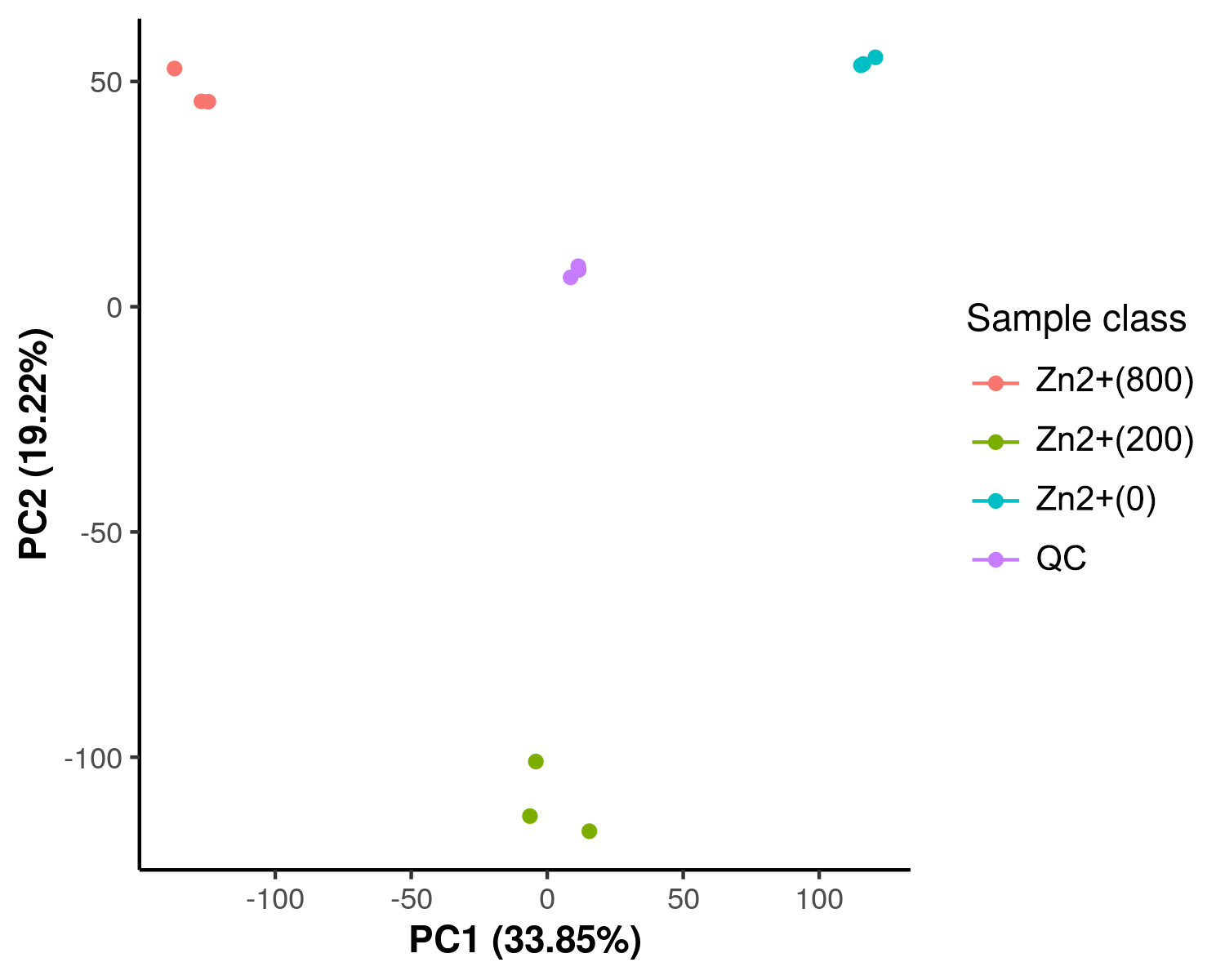

Supplement: S2 Data — (ZIP) [file pone.0295573.s002.zip › Metabonomic data/combine.pca.score.png]

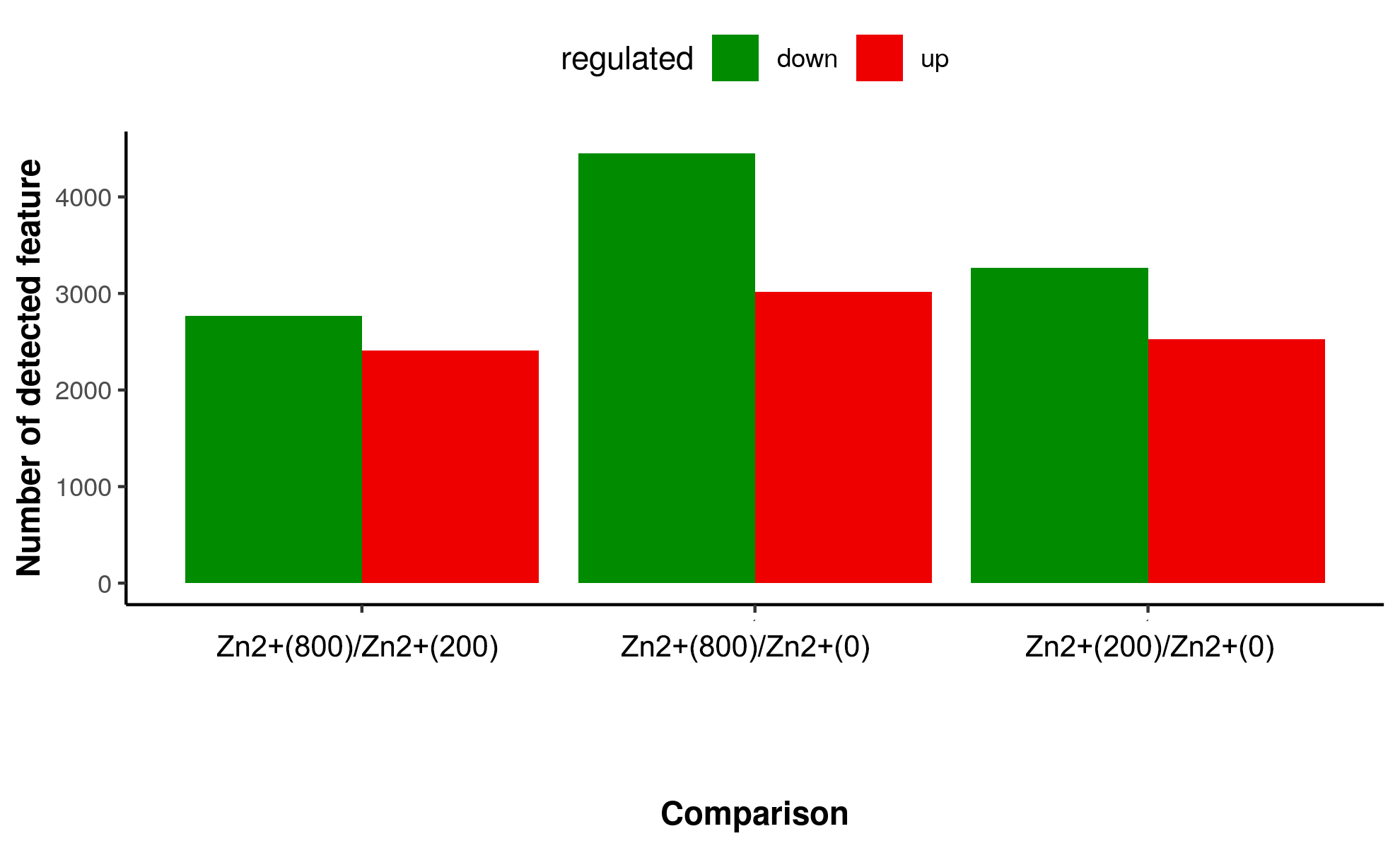

Supplement: S2 Data — (ZIP) [file pone.0295573.s002.zip › Metabonomic data/comparison.stat.png]

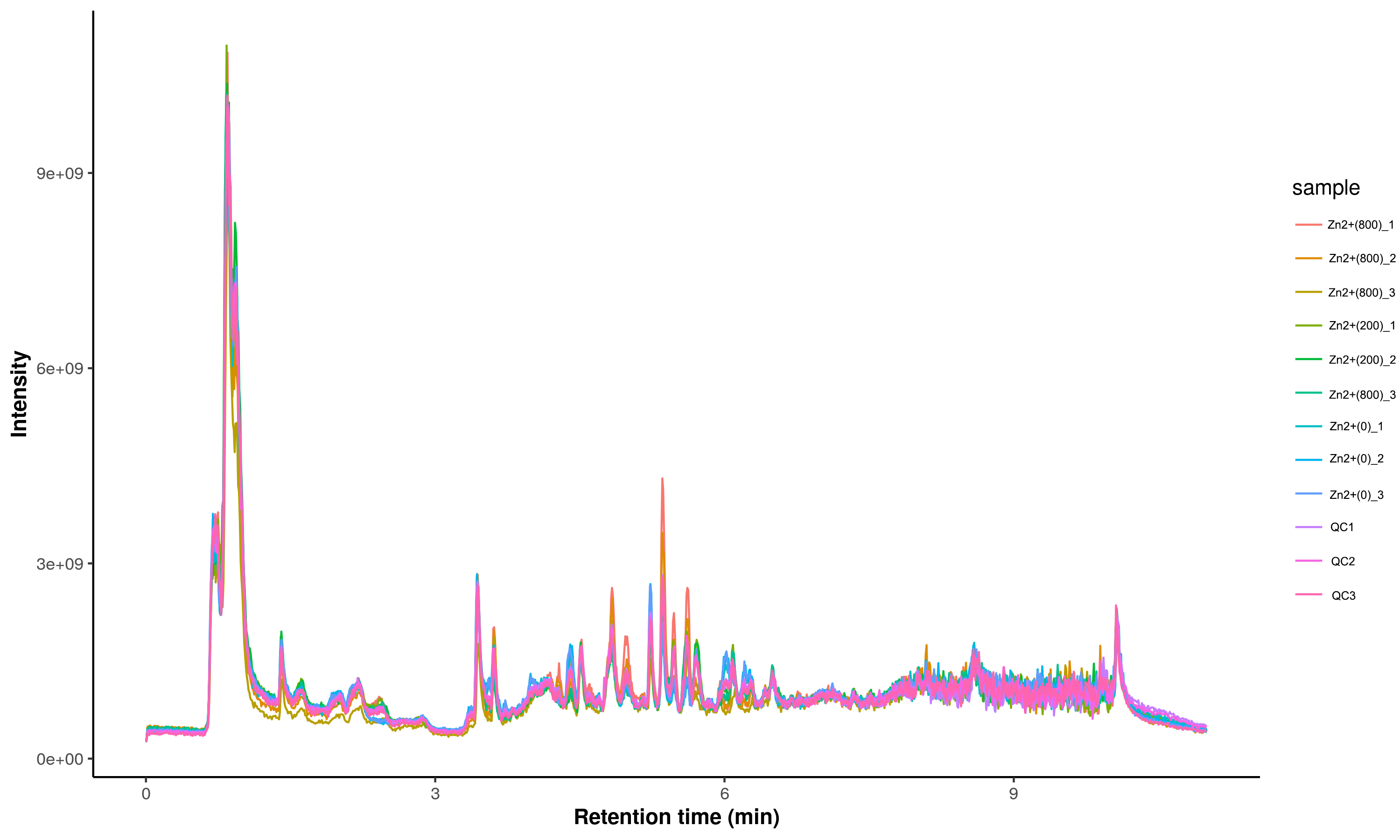

Supplement: S2 Data — (ZIP) [file pone.0295573.s002.zip › Metabonomic data/pos.tic.all.png]
