# Supplementary material for: The effects of zinc sulfate on mycelial enzyme activity and metabolites of Pholiota adiposa
Source: PLoS One. 2023 Dec 21;18(12):e0295573. doi: 10.1371/journal.pone.0295573 (PMC10735028; doi:10.1371/journal.pone.0295573)
Supplement: Attachment — Submitted filename: 1011Respond to reviewer.doc [file pone.0295573.s003.doc]

**Reviewer #1**: Pholiota adiposa, belonging to the Fungi genus, is a widely distributed edible fungus with significant industrial importance. But only question is that why do we need to treat the edible fungi with Zinc sulfate? The article is written well and presented properly so, may be considered.

**Author :** Thank you for your reviews!

Zinc is an essential micronutrient for plants and fungi. It plays a crucial role in enzymatic activities and helps in various metabolic processes. Treating fungi with zinc sulfate ensures that they have an adequate supply of this nutrient, which can promote their growth and development; Zinc sulfate treatment can help prevent certain diseases in edible fungi. Zinc has been shown to have antifungal properties, inhibiting the growth of harmful fungi that can cause diseases in mushrooms. By treating the fungi with zinc sulfate, the risk of infections and diseases can be reduced; Enhancing flavor and quality: Zinc sulfate treatment has been found to improve the flavor and quality of edible fungi. Zinc is involved in the synthesis of various compounds that contribute to the taste and aroma of mushrooms. By providing zinc to the fungi through zinc sulfate treatment, the resulting mushrooms can have a more desirable flavor and overall better quality; Increased yield: Zinc sulfate treatment has been shown to increase the yield of edible fungi. Zinc is involved in various metabolic processes, including photosynthesis and carbohydrate metabolism. By ensuring that the fungi have sufficient zinc, their overall growth and productivity can be enhanced, leading to higher yields.

**Reviewer #2**: 1. Please ensure all plants' scientific names are written appropriately ie. in italics font

**Author :** Thank you for your reviews! I have checked the full manuscript and modified it accordingly.

2. In the introduction - authors introduced about Pholiota adiposa, but later went on to highlight about Pleurotus ostreatus. Was it an unintentional typo error or could it be the way the sentence is written?

**Author :** What appears in the first paragraph of the introduction should be *Pholiota adiposa*. A noun error has occurred and has been revised.

3. Please re-check and ensure your figures and tables are numbered correctly and matches the description / discussion that follows.

for example - There are 2 figures labelled as 11A, B,C and D. Which one is the actual Fig 11?

It was also difficult to follow the discussion referred by table 3, table 4, Fig 7 as the numbering was not properly matched.

**Author :**Thank you for your reviews! The figures has been re-tagged, and there is no problem with the marking of figure 7 and tables 3 and 4.

4.For part "preparation of samples" - please explain as to why the need to grind the centrifuge tubes wall, shouldn't it be the sample?

**Author :**I am very sorry that my expression has been revised in the article, please review!

5.For 'metabonomic sample processing' - are you using buffer or just distilled water? Please remove the word buffer if it's only distilled water. And should it be buffer, please explain the type and preparation of the buffer.

**Author :** Thank you for your reviews! Buffer has been deleted.

6. Figure 5 - Heat map. Please provide a small explanation for this interesting map.

**Author :**Thank you for your reviews! The heat map is explained after ( figure 5) in ‘Enzyme activity determination’

7. Correlation analysis - based on table 2, your correlation description were unmatched. Please relook at what have been mentioned for amylase, cellulase, SOD and CAT.

**Author :**Thank you for your reviews! Correlation analysis has been recalibrated

8. Conclusion - please revise as based on the results, the effect of zinc is not in concentration dependent manner. It is instead Zn 200>0>400>800, clearly not a concentration dependent pattern.

**Author :**Thank you for your reviews! The conclusion has been revised.

9. Please do a simple language check e.g on page 14 'to explaining' suppose to be 'to explain' and on page 10 'maximum inject time' should be written as 'maximum injection time'

**Author :**Thank you for your reviews! I have modified it as required.
